# A ventral midline primary schwannoma of the cervical spinal cord: A case report

**DOI:** 10.1097/MD.0000000000021433

**Published:** 2020-10-02

**Authors:** Fengqing Gong, Yongjie Chen, Naichun Yu, Zongguang Li, Guangrong Ji

**Affiliations:** Department of Orthopedic Surgery, Xiang’an hospital of Xiamen university, Xiamen City, Fujian Province, China.

**Keywords:** cervical spinal cord, schwannoma, ventral midline

## Abstract

**Introduction:**

Intradural schwannomas can occur at any level of the spine. According to the literature, approximately 8% of intradural schwannomas occur in the atlantoaxial spine, and these tumors are usually located in the posterolateral or lateral spinal cord. In contrast, tumors in the ventral midline of the spinal cord are relatively rare.

**Patient concerns:**

A 47-year-old female presented with progressively worsening neck pain and paresthesias in both upper and lower limbs for the past 5 years.

**Diagnosis:**

Based on Magnetic Resonance Imaging and histopathological findings, she was diagnosed with ventral midline primary schwannoma of the cervical spinal cord.

**Interventions:**

The patient was treated with surgical resection.

**Outcomes:**

Follow-up visit at 2 years after the surgery showed that the patient is neurologically intact and free of disease.

**Conlusion:**

In summary, for the tumors in the ventral midline of the atlantoaxial spinal cord, the preferred treatment is complete surgical resection by the posterior approach compared to the anterior approach, which often improves clinical symptoms or achieves a healing effect.

## 1 Introduction

Primary tumors of the spinal cord account for approximately 2% to 4% of primary tumors of the central nervous system.^[1]^ According to the location of tumors, they can be divided into intramedullary, intradural extramedullary and extradural.^[2]^ Intradural extramedullary tumors are usually benign World Health Organization (grade I) and mainly comprise schwannomas, neurofibromas and meningiomas.^[3]^ Schwannomas are the most common intradural extramedullary spinal tumors, accounting for approximately 55% of intraspinal tumors.^[4]^ Intraspinal schwannomas are predominant between 40 and 60 years old, but the malignant degree of these tumors has no special relationship with age.^[1]^ Intradural schwannomas can occur at any level of the spine. According to the literature, approximately 8% of intradural schwannomas occur in the atlantoaxial spine,^[5]^ and these tumors are usually located in the posterolateral or lateral spinal cord. In contrast, tumors in the ventral midline of the spinal cord are relatively rare.^[6]^ This article reports a case of a ventral midline schwannoma in the cervical spinal canal. Additionally, the mechanism, clinical manifestations, imaging features and treatment methods of schwannomas are reviewed.

## 2 Case report

A 47-year-old female presented with progressively worsening neck pain and paresthesias in both upper and lower limbs for the past 5 years. She had progressive stiffness of all limbs, difficulties in holding and walking, straining during micturition and constipation for the past week. Her reflexes were exaggerated bilaterally in the biceps and triceps brachii in both upper limbs.

The patient also developed hypermyotonia in both the upper and lower limbs. The upper and lower limb muscle force was grade III. The Hoffmann sign in both upper limbs and the Babinski sign in lower limbs were positive. The patient had been treated for cervical spondylosis, but the symptoms were not significantly relieved.

**Figure 1:**
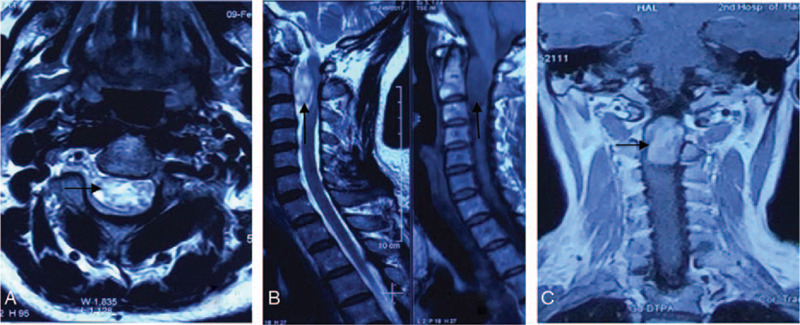
MRI of the cervical spine revealed a C1-C2 ventral midline tumor measuring 3.0 × 2.0 cm. A. T1-weighted image (Axial); B. T2-weighted image and T1-weighted image (Sagittal); C. Gadolinium enhanced MRI (Coronal). MRI = magnetic resonance imaging.

Magnetic resonance imaging (MRI) of the cervical spine revealed a C1-C2 ventral midline tumor measuring 3.0 × 2.0 cm. The tumor was large, accounting for almost 90% of the volume of the spinal canal, and the spinal cord was severely compressed and displaced posteriorly (Fig. 1A). The tumor was hypointense on T1-weighted images and hyperintense on T2-weighted images (Fig. 1B), with uneven enhancement on a Gdolinium diethylenetriamine pentaacetic acid enhanced scan (Fig. 1C).

**Figure 2:**
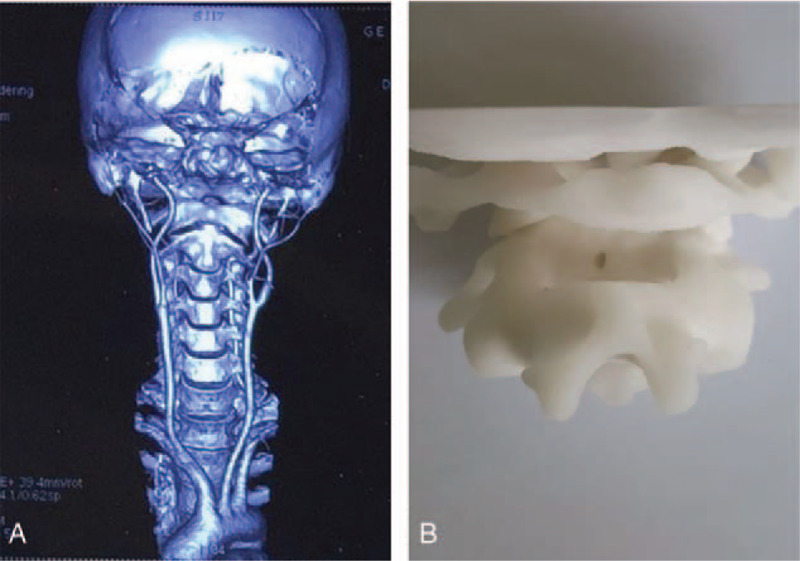
A. Three-dimensional CTA of the cervical spine shows the relationship between blood vessels and the vertebral spine; B. Printing the atlantoaxial spine shows the anatomy of the upper cervical spine. CTA = computed tomography angiography.

Preoperatively, we demonstrated the relationship between blood vessels and the vertebral spine by 3-dimensional Computed Tomography Angiography of the cervical spine (Fig. 2A). Moreover, the anatomy of the upper cervical spine was examined by printing the atlantoaxial spine with 3-dimensional printing technology (Fig. 2B), which could provide a more accurate preoperative plan for the surgeon such as the position, diameter, and length of the pedicle screw and so on, and improve the safety of the operation.

**Figure 3:**
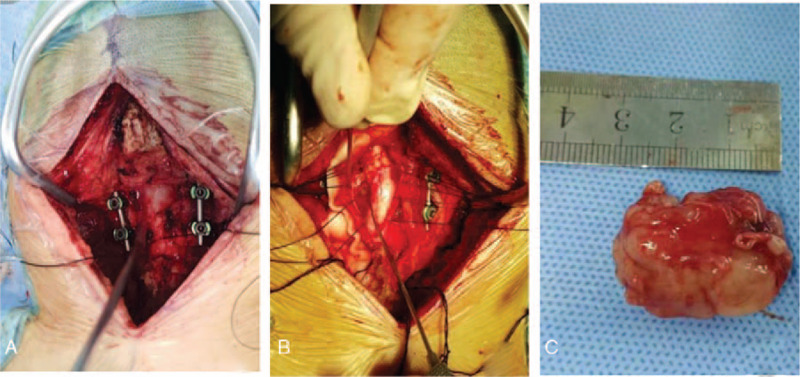
A. After the atlantoaxial fixation was fixed, the dura was exposed. B. The tumor was carefully separated from the spinal cord. C. The tumor was completely removed with the peripheral fibrous capsule.

First, the posterior structure of the atlantoaxial spine was exposed, and pedicle screw fixation of the atlantoaxial spine was designed according to the preoperative three-dimensional printing model to ensure the stability of the atlantoaxial spine (Fig. 3A). Sufficient decompression was performed during the operation, including the posterior spinal canal osseous structure and longitudinal incision of the dura mater. The tumors were carefully separated from the spinal cord, and the nerve fibers carrying the tumors were clearly severed (Fig. 3B). The ventral tumor of the spinal cord was completely removed with the periphery fibrous capsule (Fig. 3C). The dura mater was sutured, and the vital signs of the patient were stable during the operation.

**Figure 4:**
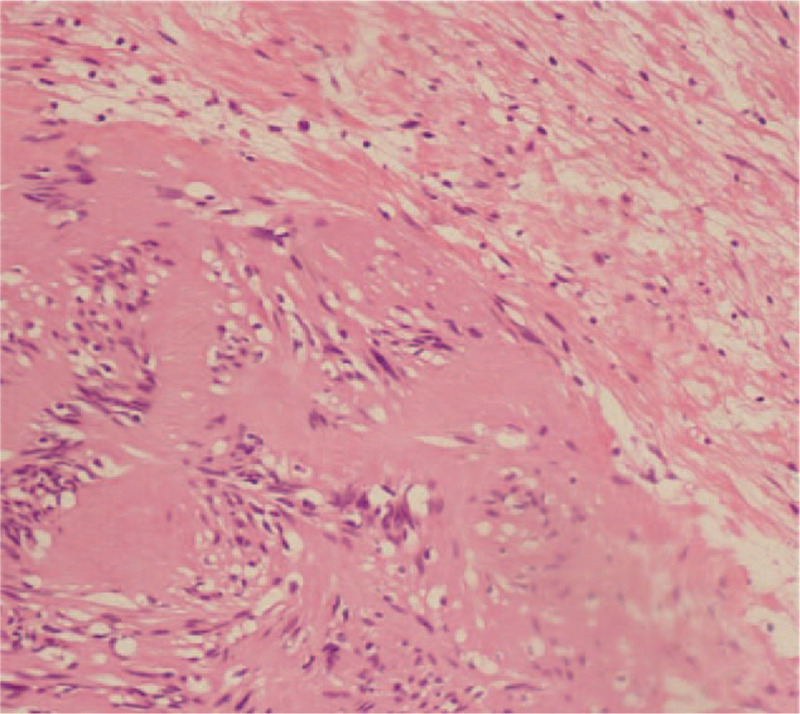
Hematoxylin-eosin (HE) stained photomicrograph (original magnification ×200) of the specimen showing the Antoni A areas (consisting of short, bundled shuttle-shaped cells, with a narrowed nucleus at 1 end, abundant cytoplasm, faint irritated red, and unclear cell boundary) and Antoni B areas (cells are sparse and loosely arranged).

**Figure 5:**
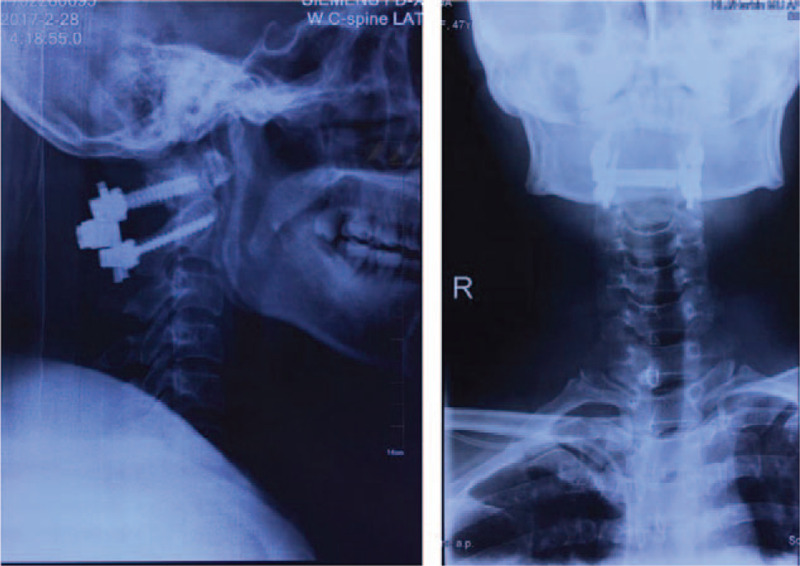
X-ray showing that the cervical spine sequence was stable, and the internal fixation position was good.

**Figure 6:**
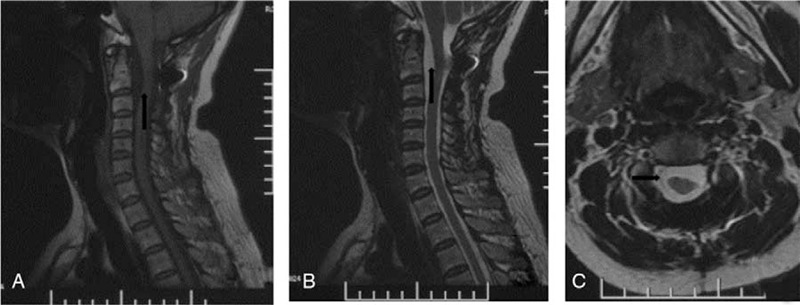
MRI showing that the no recurrence of the tumor 2 years postoperatively. MRI = magnetic resonance imaging.

The histopathological examination of the specimen showed features of a schwannoma (Fig. 4A, B). Postoperatively, the patient's nerve function recovered well, and her limbs moved freely. X-ray imaging showed that the cervical spine sequence was stable, and the internal fixation position was good (Fig. 5A, B) The MRI showed no recurrence of the tumor 2 years postoperatively (Fig. 6A, B,C).

Patient has provided informed consent for publication of the case.

## 3 Discussion

Intraspinal schwannomas and neurofibromas are classified as spinal nerve sheath tumors,^[7]^ which are categorized as neurofibromatosis (NF) and type II (NF2). Schwannomas are more common in NF2. Ninety percent of schwannomas are isolated, and 4% of schwannomas are associated with NF2, while the remaining 5% of schwannomas are varied but not related to NF2.^[8]^ The incidence of NF2 is low, approximately 1/25000–40000, and NF2 is often accompanied by bilateral vestibular schwannomas.^[9]^ NF2 is associated with more aggressive biological behavior of schwannomas, and all young patients with NF2 often develop multiple schwannomas with a higher risk of malignant transformation. Fortunately, this patient had an isolated schwannoma. Schwannomas mainly originate from the dorsal nerve, but 23% of cervical schwannomas originate from the ventral nerve roots.^[10]^ All intraspinal schwannomas are mostly located in the dorsal or anterolateral side of the spinal cord, but in this article, the tumor was in the ventral midline of the spinal cord. This finding suggests an additional origin location for schwannomas. In the literature, some hypotheses are proposed for the mechanism of ventral schwannoma in the spinal cord:

(1) from the anterior spinal artery through the perivascular vascular plexus of the spinal cord,[^11^,^12^,^13^,^14^]
(2) improper neural crest progenitor cell migration to the central nervous system parenchyma during the embryonic period.[^11^,^13^,^15^]

Although schwannomas have a malignant subtype, they are still considered benign tumors. Spinal canal schwannomas can occur at any level of the spine, and the incidence increases from the high neck to the thoracolumbar region. This growth pattern is related to the following anatomical feature: the nerve root at the distal end of the spine has a longer intradural composition.^[5]^ Moreover, according to the site, tumors can be divided into intradural extramedullary, extradural, intra-extradural (dumbbell) and intramedullary tumors.^[5]^ Based on previous reports, 70% to 80% of intraspinal schwannomas occur in the dura, and approximately 15% in occur other sites. Intramedullary schwannomas are very rare.^[5]^

Regarding clinical manifestations, patients may not have any symptoms at the beginning of their illness. Most patients present with mild neuroradial pain or paresthesia. Some patients may develop spontaneous pain, but this issue is uncommon. Tumors in most patients are accidentally discovered when considering radiculopathy or spinal cord disease. This patient had been treated conservatively for cervical spondylosis; however, the symptoms were not significantly relieved and continued to worsen. Ultimately, lesions occupying the spinal canal were uncovered after MRI. Therefore, MRI is preferred in the diagnosis of schwannomas. Because the vertebral body is not involved in the early stage of the tumor, it is difficult to find on X-ray, and CT scans are not as clear as MRI scans. On MRI, schwannomas usually appear as solid tumors that grow in the dorsal root zone, showing the displacement of spinal cord, conus medullaris or filum terminale.^[3]^ Schwannomas are usually isointense on T1WI and hyperintense on T2WI, and contrast enhancement is diminished when cystic lesions are present. Meningiomas are often isointense and hyperintense on T2WI.^[16]^ We often use this technique to identify these tumors. Since schwannomas do not usually encompass hemorrhage or calcification, there is no obvious feature on MRI that can be differentiated from NF.

For schwannomas treatment, if there are no clinical symptoms, we can select conservative treatment and check MRI regularly, considering the benign biological manifestations. For schwannomas with clinical signs or imaging signs of enlargement, surgical resection is generally performed. Surgical resection can improve clinical symptoms or achieve a healing effect.^[17]^ Although the surgical tumor in this case was located in the middle of the ventral side of the spinal cord, the anterior approach has considerable problems such as a deep field of view and insufficient exposure, excessive epidural venous plexus hemorrhage, inability to ensure postoperative spinal stability and postoperative cerebrospinal fluid leakage.^[18]^ We used the posterior approach traditionally^[19]^ and operated as far as possible from the lateral side of the spinal cord. Additionally, the posterior arch of the atlas and the vertebral lamina of the axis were removed, and the lateral block joints on the left side of the pivotal axis were removed to reduce the irritation to the spinal cord. To ensure the stability of the atlantoaxial spine, the pedicle screw fixation of the atlantoaxial spine was performed firstly. Then the use of drugs such as hormones and dehydration during slow removal of tumors reduces the risk of ischemia-reperfusion injury and spinal cord edema after tumor removal. The patient's postoperative neurological function recovered well, and the limbs were normal. For patients with incomplete tumor resection or malignant schwannomas, postoperative radiotherapy has also achieved satisfactory results,^[20]^ but the risk associated with radiation therapy needs to be communicated to patients. Furthermore, most patients need long-term follow-up after surgery to prevent tumor progression or recurrence.

## 4 Conclusion

In summary, the tumors in the ventral midline of the atlantoaxial spinal cord are relatively rare in contrast to the posterolateral or lateral spinal cord, the preferred treatment is complete surgical resection by the posterior approach compared to the anterior approach, which often improves clinical symptoms or achieves a healing effect. At the same time, adequate preoperative preparation will maximum improve the safety and the success of the surgery.

## Acknowledgments

The authors thank the members of their colleagues for making this case possible. All authors confirm that the content has not been published elsewhere and does not overlap with or duplicate their published work.

## Author contributions

**Conceptualization:** Guangrong Ji, Fengqing Gong.

**Investigation:** Fengqing Gong, Yongjie Chen

**Methodology:** Guangrong Ji.

**Resources:** Guangrong Ji, Naichun Yu, Zongguang Li.

**Supervision:** Guangrong Ji, Naichun Yu, Zongguang Li.

**Writing – editing:** Guangrong Ji, Naichun Yu, Zongguang Li.

**Writing – original draft:** Fengqing Gong

## References

[1] Campello C Le Floch A Parker F Neuroepithelial intra medullary spinal cord tumors in adults. Study of 70 Cases[M] 2009;A33–133.

[2] Elsberg CA Some aspects of the diagnosis and surgical treatment of tumors of the spinal cord: with a study of the end results in a series of 119 operations. Ann Surg 1925;81:1057–73. 17865272 10.1097/00000658-192506010-00003 PMC1400102

[3] Abul-Kasim K Thurnher MM Mckeever P Intradural spinal tumors: current classification and MRI features. Neuroradiology 2008;50:301–14. 18084751 10.1007/s00234-007-0345-7

[4] Hirano K Imagama S Sato K Primary spinal cord tumors: review of 678 surgically treated patients in Japan. A multicenter study. Eur Spine J 2012;21:2019–26. 22581192 10.1007/s00586-012-2345-5 PMC3463691

[5] Jinnai T Koyama T Clinical characteristics of spinal nerve sheath tumors: analysis of 149 cases. Neurosurgery 2005;56:510–5. 15730576 10.1227/01.neu.0000153752.59565.bb

[6] Mahore A Chagla A Goel A Giant ventral midline schwannoma of cervical spine: agonies and nuances. J Korean Neurosurg Soc 2010;47:454–7. 20617092 10.3340/jkns.2010.47.6.454 PMC2899034

[7] Ramamurthi B Anguli VC Iyer CG A case of intramedullary neurinoma. J Neurol Neurosurg Psychiatry 1958;21:92–4. 13539649 10.1136/jnnp.21.2.92 PMC497301

[8] Antinheimo J Sankila R Carpen O Population-based analysis of sporadic and type 2 neurofibromatosis-associated meningiomas and schwannomas. Neurology 2000;54:71–6. 10636128 10.1212/wnl.54.1.71

[9] Koontz NA Wiens AL Agarwal A Schwannomatosis: the overlooked neurofibromatosis? AJR Am J Roentgenol 2013;200:W646–53. 23701098 10.2214/AJR.12.8577

[10] El-Mahdy W Kane PJ Powell MP Spinal intradural tumours: part I--extramedullary. Br J Neurosurg 1999;13:550–7. 10715722 10.1080/02688699943042

[11] Adelman LS Aronson SM Intramedullary nerve fiber and Schwann cell proliferation within the spinal cord (schwannosis). Neurology 1972;22:726–31. 4673254 10.1212/wnl.22.7.726

[12] Russell D S, Rubinstein L J. Pathology of tumors of the nervous system. 5th ed. Baltimore, md[M]. 1989: 809-854.

[13] Singer RJ Clough J Johnson M Pigmented schwannoma of the ventral spinal cord. South Med J 1999;92:532–4. 10342906 10.1097/00007611-199905000-00019

[14] Darwish BS Balakrishnan V Maitra R Intramedullary ancient schwannoma of the cervical spinal cord: case report and review of literature. J Clin Neurosci 2002;9:321–3. 12093146 10.1054/jocn.2001.0952

[15] Solomon RA Handler MS Sedelli RV Intramedullary melanotic schwannoma of the cervicomedullary junction. Neurosurgery 1987;20:36–8. 3808271 10.1227/00006123-198701000-00010

[16] Gu R Liu JB Zhang Q MRI diagnosis of intradural extramedullary tumors. J Cancer Res Ther 2014;10:927–31. 25579530 10.4103/0973-1482.137993

[17] Conti P Pansini G Mouchaty H Spinal neurinomas: retrospective analysis and long-term outcome of 179 consecutively operated cases and review of the literature. Surg Neurol 2004;61:34–43. 14706374 10.1016/s0090-3019(03)00537-8

[18] O’toole JE Mccormick PC Midline ventral intradural schwannoma of the cervical spinal cord resected via anterior corpectomy with reconstruction: technical case report and review of the literature. Neurosurgery 2003;52:1482–5. 12762896 10.1227/01.neu.0000065182.16584.d0

[19] Mccormick PC Post KD Stein BM Intradural extramedullary tumors in adults. Neurosurg Clin N Am 1990;1:591–608. 2136160

[20] Monserrate A Zussman B Ozpinar A Stereotactic radiosurgery for intradural spine tumors using cone-beam CT image guidance. Neurosurg Focus 2017;42:E11. 10.3171/2016.9.FOCUS16356 28041317

